# Men’s Preference for Women’s Facial Features: Testing Homogamy and the Paternity Uncertainty Hypothesis

**DOI:** 10.1371/journal.pone.0049791

**Published:** 2012-11-21

**Authors:** Jeanne Bovet, Julien Barthes, Valérie Durand, Michel Raymond, Alexandra Alvergne

**Affiliations:** 1 University of Montpellier II, Montpellier, France; 2 Institute of Evolutionary Sciences, Center for Scientific Research, Montpellier, France; 3 Department of Animal and Plant Sciences, University of Sheffield, Sheffield, United Kingdom; Universidad Carlos III de Madrid, Spain

## Abstract

Male mate choice might be based on both absolute and relative strategies. Cues of female attractiveness are thus likely to reflect both fitness and reproductive potential, as well as compatibility with particular male phenotypes. In humans, absolute clues of fertility and indices of favorable developmental stability are generally associated with increased women’s attractiveness. However, why men exhibit variable preferences remains less studied. Male mate choice might be influenced by uncertainty of paternity, a selective factor in species where the survival of the offspring depends on postnatal paternal care. For instance, in humans, a man might prefer a woman with recessive traits, thereby increasing the probability that his paternal traits will be visible in the child and ensuring paternity. Alternatively, attractiveness is hypothesized to be driven by self-resembling features (homogamy), which would reduce outbreeding depression. These hypotheses have been simultaneously evaluated for various facial traits using both real and artificial facial stimuli. The predicted preferences were then compared to realized mate choices using facial pictures from couples with at least 1 child. No evidence was found to support the paternity uncertainty hypothesis, as recessive features were not preferred by male raters. Conversely, preferences for self-resembling mates were found for several facial traits (hair and eye color, chin dimple, and thickness of lips and eyebrows). Moreover, realized homogamy for facial traits was also found in a sample of long-term mates. The advantages of homogamy in evolutionary terms are discussed.

## Introduction

Women’s facial attractiveness reflects features that indicate high reproductive potential (for a review in humans, see [1]). For example, feminine characteristics are strong components of women’s attractiveness and are linked to estrogen/androgen ratios–a reliable indicator of fertility potential [2]. However, some facial features, such as eye or hair color or dimples, do not exhibit any apparent association with individual fitness. These so-called “neutral features” have been proposed to be subject to sexual selection and are specifically linked to paternity confidence [3]. In this context, these traits are considered attractive because they increase the probability that paternal traits are expressed in the phenotype of the offspring, thereby facilitating the detection of paternity or non-paternity. Another hypothesis for the role of these facial features in mate choice is homogamy, where individuals demonstrate preferences for mates with similar genetic backgrounds [4], [5], [6]. In fact, mating with a partner of relatively proximal genetic background might be evolutionary advantageous by decreasing outbreeding depression. In this study, we aimed to simultaneously investigate the role of paternity confidence and homogamy in influencing the attractiveness of women’s facial features.

In species where paternal care is required for the survival of the offspring, uncertain paternity might cause males to distinguish their own offspring from those conceived by their mate in extra-pair relationships [7]. In humans, some studies have suggested that men are sensitive to cues of paternity when making decisions of paternal investment, such as fidelity and father-child facial similarities (in experiments [8], but see [9]; in real families [10], [11], [12], [13], [14]). If men assess their paternity through phenotype matching [15], then the phenotypic traits exhibiting Mendelian or quasi-Mendelian inheritance could be used to facilitate the detection of paternity or non-paternity. In this context, men displaying recessive features would demonstrate a preference for women exhibiting recessive versions of the same features, because their children will be phenotypically recessive for the trait, and a phenotypically dominant child could then signal a non-paternity. On the other hand, a phenotypically dominant man may conceive, with some probabilities, phenotypically dominant or recessive children, irrespective of the phenotype of his partner (the probabilities depend, among other, on allele frequencies in the population). Nevertheless, choosing a phenotypically recessive woman increases the probability that his dominant traits (rather than her recessive traits) will be expressed by their offspring, confirming transmission of paternal features (this probability is at least 0.5). As the number of dominant traits considered increases, the probability that at least one of them is transmitted also increases. Thus, the paternity uncertainty hypothesis predicts that both phenotypically dominant and recessive men prefer phenotypically recessive women, but this preference may be more important for recessive men.

An empirical evaluation of how preferences vary relative to one’s own genetic determinism is available for eye color. Blue eye color is recessive to brown [16], [17], [18], [19], and interestingly, a study has found that blue-eyed men tend to find blue-eyed women more attractive, whereas brown-eyed men were not found to exhibit a particular eye color preference [20] but see [21]. This result is consistent with eye color influencing attractiveness through a mechanism of enhanced non-paternity detection. This suggests that so-called neutral traits–in this case, eye color–might be subject to sexual selection. However, other unknown reasons (other than sexual selection) underlying eye color selection cannot be definitively excluded. Moreover, unless other polymorphic traits are also considered, the paternity uncertainty hypothesis cannot be validated. Indeed, the reliability of paternity confidence evaluation should increase with the number of traits evaluated. This is because the dominance effect blurs the relationship between phenotype and genotype, depending on the population frequency of heterozygotes. In particular, non-paternity detection using eye color is less reliable in a population where the majority of the population exhibits the same eye color, as there will be a high probability that the biological and the social fathers have the same eye color. Thus, when several dominant traits are simultaneously involved, the probability increases that at least one of them is transmitted and signals paternity.

Another hypothesis about the role of these so-called neutral facial features in mate choice is homogamy [4], [5], [6]. The extent to which there is selection for homogamy remains poorly understood and could result from an extension of a kin selection mechanism, as a lower genetic distance between parents translates into a higher parental-offspring relatedness (Genetic similarity theory, [22] or outbreeding avoidance [23]). Indeed, a majority of mates resemble each other for a high number of traits. Positive correlations have been demonstrated for socioeconomic, psychological and physical variables [5], [6], [24], [25]. Nevertheless, the observed formation of couples does not always reflect preferences. For example, if mate choice occurs within a given socioeconomic class [26], [27], [28], it will automatically generate homogamy for specific factors of each class. Competition for mates might also automatically generate homogamy for some features, such as attractiveness [29].

To simultaneously evaluate the paternity uncertainty (preferences for recessive states of features) and the homogamy (homogamy for facial traits independent of their dominance/recessive status) hypotheses, experiments were performed in which men were asked to rate the attractiveness of women’s facial pictures. The dominance/recessive status of several facial traits was recorded for both women and raters. In Study 1, facial pictures from real women were used to investigate (i) whether raters prefer women with recessive facial features (i.e., paternity uncertainty hypothesis) and (ii) whether raters prefer women whose features are similar to their own, independently of the genetic determinism of these traits (i.e., homogamy hypothesis). In Study 2, the same test was also performed using artificial faces as stimuli to better control for the large number of dimensions involved in attractiveness. In all studies, these traits were first analyzed independently in the event of differing results, and then were simultaneously considered in order to provide an overall answer independent of specific traits. Finally, in Study 3, data derived from real couples were analyzed to evaluate whether the preferences previously identified are applicable to the formation of couples.

## Materials and Methods

### Ethics Statement

The protocol used to recruit participants and collect data was approved (#1226659) by the French National Committee of Information and Liberty (CNIL). For each participant, the general purpose of the study was explained (“a study on the determinants of facial attractiveness”) and a written voluntary agreement was requested for a statistical use of data (private information and photographs). Data were analyzed anonymously.

### Study 1

#### Stimuli

**Figure 1:**
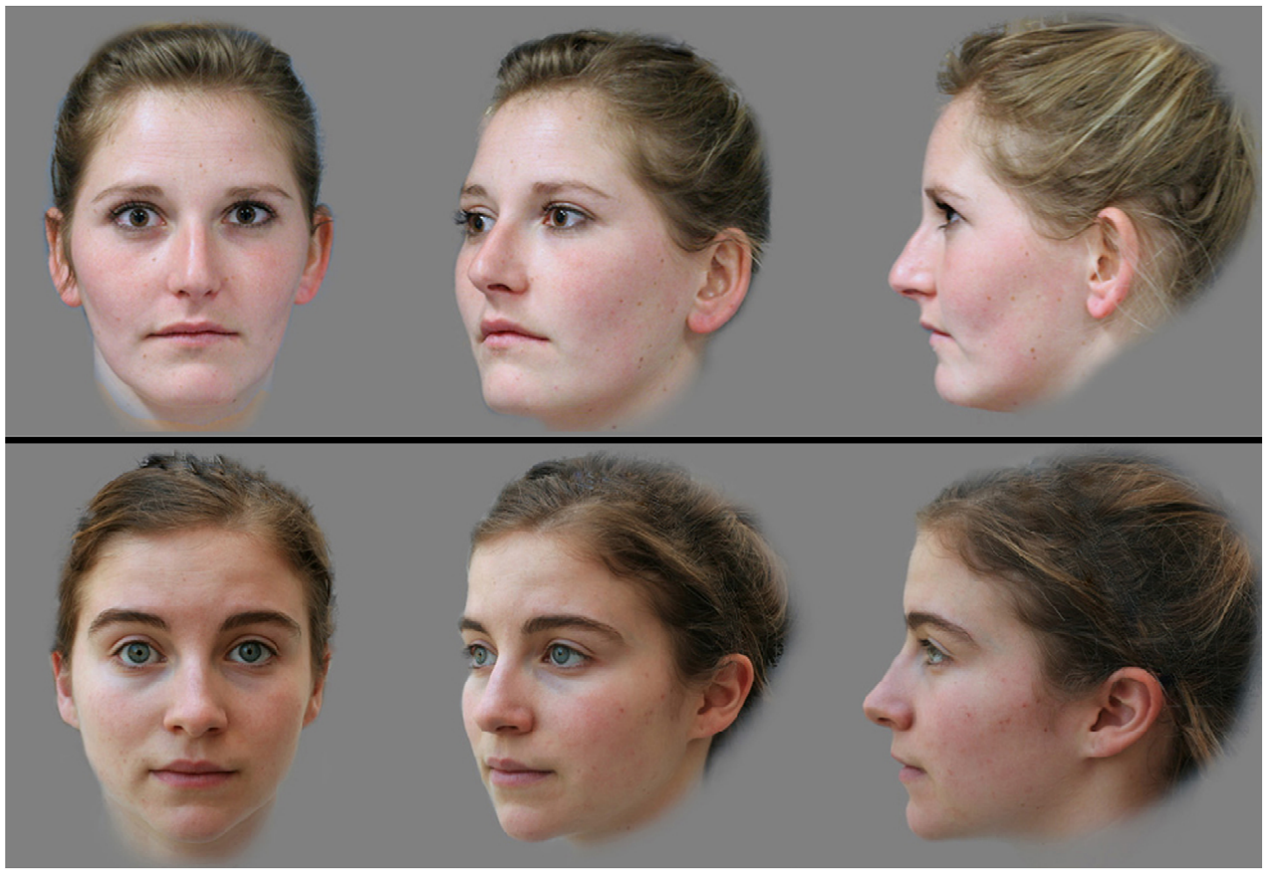
A typical screen shot during the evaluation of the women’s facial features by the raters in Study 1. Women aged 18–25 were recruited and three facial photographs (triptych) were taken. A computer program was used to randomly present drawn pairs of triptychs to male raters. For each pair, the rater had to click on the picture depicting the woman that he found the most attractive. The pictures and information were used with each woman’s consent for publication.

Women between 18 and 25 years of age were recruited by social networks and in public places from Montpellier, Pau and Reims in France. For each woman, the following information was collected: place and date of birth, origin of parents and grandparents, socioprofessional category, monthly income (divided into nine classes from less than 760 € to more than 3618 €), and level of education. Three facial photographs (front, profile and three quarter views) were taken with the same digital still camera (Canon EOS 20D) at a distance of 1 m using the same general setting. Each individual was asked to express a neutral face and to remove any glasses or earrings. All photographs were electronically processed using Adobe Photoshop CS2 to normalize size, color balance, contrast and luminosity. The backgrounds were replaced by a uniform grey color. Each set of three facial views was assembled in a triptych (see figure 1).

#### Procedures

A Delphi-based computer program was generated to present randomly drawn pairs of triptychs to male raters (see figure 1). For each pair, the rater had to click on the picture depicting the woman he found the most attractive. The position of the picture on the screen (up or down) was randomly ascribed. A given rater had 20 distinct pairs of pictures to assess, corresponding to 40 different women. Three pairs, randomly chosen among those previously seen, were presented again at the end to estimate judgment reliability. Male volunteer raters were recruited in public places in Montpellier, France and were unaware of the purpose of the study when assessing pairs of women. For each rater, the following information was collected: date and place of birth, origins of parents and grandparents, monthly income, occupation, house ownership, taxability, education level and sexual orientation. After completing the evaluation of women’s attractiveness, three facial photographs (front, profile and three quarter views) were taken for each rater using the same camera and conditions as above. The general purpose of the study was then explained (“a study on the determinants of facial attractiveness”) and a written agreement was requested to use the data (private information and the photographs).

#### Facial traits

A total of 16 variable facial traits with a quasi-Mendelian inheritance [17], [18], [19], [30], [31], [32], [33] were first considered. These features corresponded to various morphological features of the ear, eye, eyebrows, nose, chin, hair, mouth, skin and skull. These traits were classified among 400 triptychs as recessive or dominant by three independent evaluators (JB, MR and AA). Congruent scoring was statistically tested with a nonparametric Kappa test of concordance (K). Among congruent scores, seven features that were sufficiently frequent (>7%) in the studied population were selected for our study as follows: hair and eye color (dark or light, hair: K = 0.48, *P*<0.001; eye: K = 0.85, *P*<0.001), hair texture (curly or flat, K = 0.37, *P*<0.001), chin dimple (presence or absence, K = 0.50, *P*<0.001), thickness of lips and eyebrows (thick or thin,lips: K = 0.55, *P*<0.001; eyebrows: K = 0.57, *P*<0.001), and earlobe (attached or free, K = 0.65, *P*<0.001). According to their known inheritance pattern, the states of these features were considered here as discrete variables, although this is an approximation due to the fact that 1) the exact genetic determinisms are not fully known, 2) there is a partial environmental influence [34], and 3) some traits (e.g. lips thickness) display a unimodal distribution when quantitatively measured (unpublished results). These seven traits were typed for all female participants and male raters.

#### Morphological measurements

Twenty-one points of interest were positioned on the front face photograph of each female participant [35], [36] (see figure S1). Differences between 8 corresponding distances on each side of the face were calculated for each woman. Each difference was weighted by the mean of the two corresponding distances. A Principal Component Analysis (PCA) was performed on the resulting four variables to obtain a new synthetic variable corresponding to an asymmetry axis. The coordinate of each woman along this axis represents her individual asymmetry synthetic value. To assess femininity, 9 distances were computed from the same twenty-one points for all women subjects. Similar measurements were performed on a random sample of sixty males (derived from the raters). A linear discriminant analysis (LDA) according to sex was performed on the male and female measurements, providing a synthetic variable corresponding to a masculinity/femininity axis. The coordinate of each woman along this axis represents her individual femininity value (see figure S2). The contrast between lip and skin color was also measured. Pictures were converted to black and white, and grey values were measured for four points on the lips and four points on skin around the mouth. The difference between the means of the grey values of the lips and skin was used as a contrast value. All morphological measurements were performed using Image J software, version 1.43.

#### Statistical analysis

Logistical regression was used to analyze raters’ preferences. The response variable corresponded to being chosen or not for each woman during the presentation of each pair (binary variable). Women and raters were considered random samples from a larger population of interest and were thus random-effect variables. Therefore, generalized linear mixed models with binomial error structure were used. A recessivity index was created that varied from 0 (no recessive trait displayed by the woman) to 7 (all traits considered displayed the recessive variant by the woman). For each choice made by a rater, the difference between the recessivity indices of the focal and the non-focal woman was calculated. The value of this difference (variable “recessivity”) was integrated into the models. Then, a homogamy index was created. For each woman, the number of traits for which a common state (recessive or dominant) was shared with the rater was scored. The difference between the two women subjects for this score was entered into the model (variable “homogamy”). This score varied from 7 (the focal woman exhibited all traits in common with the rater whereas the non-focal exhibited none) to −7. To control for potential confounding effects, other variables were included in the models. These variables included the following factors: the rater’s age, and socioeconomic status (a PCA combination of the variables “monthly income”, “occupation”, “house ownership”, “taxability” and “education level”), as well as variables affecting women’s attractiveness, such as fluctuating asymmetry, degree of femininity, lip contrast and age. For asymmetry and femininity, non-linear adjustments were explored. Because in some photographs the women subjects displayed a perceptible smile, a qualitative variable describing this aspect (absent, slightly perceptible, perceptible) was also introduced as a potential confounding effect on attractiveness. Indices of homogamy and recessivity were also calculated for each trait independently. These 7 binary variables were used in a model where only significant confounding variables of the previous model were added (the age of the raters and of the women subjects, smile, asymmetry and femininity). The data were analyzed by multimodel inference [37], [38]. The fits of all possible models were compared using AIC, allowing their Akaike weights to be calculated (R package MuMin). Model averaging was performed on all models, weighting the contribution of each model according to its Akaike weight [39]. The relative importance of each variable (the sum of the Akaike weights of the models in which the variable appears) and the averages of estimates (weighted by Akaike weights) were calculated. All statistical analyses were performed using R 2.14.0 software (R Core Development Team, 2005).

### Study 2

#### Stimuli

A total of 15 distinct virtual faces were created. FaceGen software permits to create artificial 3D faces varying for general face shape, lips and eyebrows (width, thickness, color), and expression (smile). Different hair styles and color (dark or light) were added with TAAZ software. Using Photoshop, eye color (dark or light) and chin dimple were modified. Variations of the five traits under study (see below) were within the normal range of variation displayed by stimuli of Study 1 (details not shown).

#### Procedure

**Figure 2:**
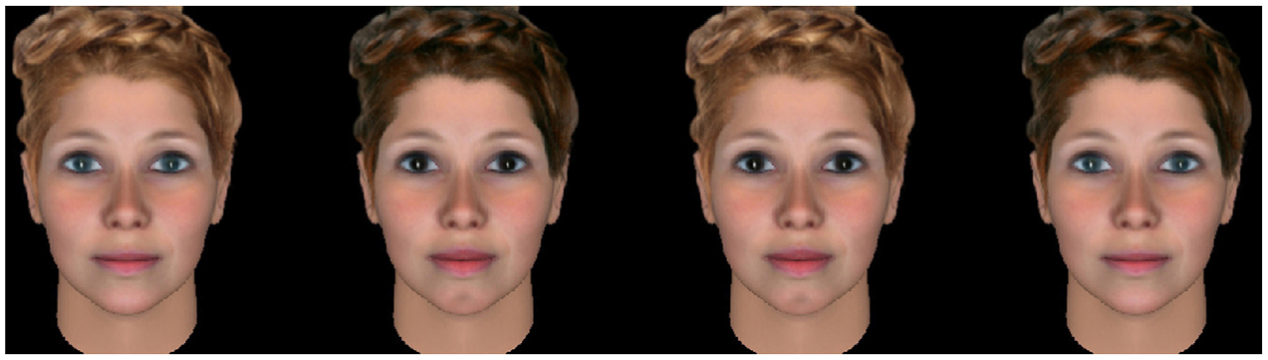
An example of a quartet used in Study 2. A Delphi-based computer program was used to present the virtual women’s faces. For a given rater, four faces of the same virtual woman were selected, depending on the rater’s own traits (initially recorded in the computer program by the observer; see table 1). These four faces corresponded to the four cases of preferences: homogamy (the face displayed similar character states as the rater), heterogamy (the face displayed opposite character states), recessivity (the face displayed recessive character states) and dominant (the face displayed dominant character states). The rater was instructed to click on the woman that he would prefer to build a family with. Images are from virtual subjects.

For each rater, the same information as in Study 1 was collected. During a preliminary questionnaire, an observer recorded the state of the five traits (hair and eye color, chin dimple, and thickness of lips and eyebrow) from the rater’s face. A Delphi-based program was used to present the virtual women’s faces. For a given rater, four faces (a ‘quartet’; see figure 2) were selected, depending on the rater’s own traits that were initially recorded in the computer program by the observer (see table 1). Within a quartet, only 5 features of interest vary: hair and eye color (dark/light), chin dimple (present/absent), and thickness of lips and eyebrows (thick/thin). Other characteristics (shape of the face, nose, expression, hair style), vary between quartets. The four faces corresponded to the four cases of preferences: homogamy (the face displayed similar character states as the rater), heterogamy (the face displayed opposite character states), recessivity (the face displayed recessive character states) and dominant (the face displayed dominant character states). These four faces were presented in a row, in a random order. The rater was instructed to click on the woman subject that he would prefer to build a family with. Among the five features studied, three are generally considered to be independent of women’s attractiveness (hair and eye color and chin dimple) and two are known to be femininity or attractiveness cues (“lips” and “eyebrows”; thick eyebrows are considered as more masculine and thick lips as more attractive [1]). To control for the effect of lips and eyebrows in influencing perceived attractiveness, raters were randomly separated into two groups. The first group was presented faces varying for eyes, hair, chin dimple and eyebrows (the state of the lips was in this case randomized between the quartets but constant within each quartet). The second group was presented faces varying for eyes, hair, chin dimple and lips. Each rater evaluated fifteen quartets, in a random order. To estimate judgment reliability, two quartets randomly chosen among those previously seen were presented at the end. Caucasian male volunteers were recruited in public places in Montpellier, France and were unaware of the purpose of the study when assessing quartets of women. A written agreement was obtained for usage of their private information.

**Table 1:** Example of facial features for the four women appearing in a quartet in Study 2.

	Facial features				
Women (hypothesis)	Eyes	Chin dimple	Hair	Eyebrows	Lips
Woman 1 (homogamous)	light	no	dark	thick	thin
Woman 2 (heterogamous)	dark	yes	light	thin	thick
Woman 3 (recessivity)	light	no	light	thin	thin
Woman 4 (dominance)	dark	yes	dark	thick	thick

The rater’s traits were recorded in the computer program. Then, the four faces of the same virtual woman were presented, depending on the rater’s own traits (see figure 2). An example for a rater with light eyes, no chin dimple, dark hair, thick eyebrows and thin lips.

#### Statistical analysis

The response variable was the preferred face of each rater. This variable included four modalities (homogamy, heterogamy, recessivity and dominant). Artificial faces and raters were considered random samples from a larger population of interest, and thus, both groups were considered to be random-effect variables. However, no multinomial model with random-effects was available with R 2.14.0 software. To overcome this limitation, two approaches were used. First, one response by each rater was randomly sampled, and a multinomial model was applied. The process was repeated 1,000 times, generating a distribution of *P*-values. Second, the data were analyzed by multimodel inference as in Study 1. One of the four positions was arbitrarily chosen (the first on the left), and the variable responses reflected the choice of each rater on that position (yes if chosen, no if otherwise). As in Study 1, confounding variables were added to test the effects of the raters’ characteristics (age and socioeconomic status) on their choices. Next, the homogamy and recessive indexes were calculated as in Study 1. We created overall indices for all of the traits together, plus indices for each characteristic independently. The fits of all possible binomial generalized linear mixed models were compared, and the relative importance and the weighted average of the estimates for each variable were calculated as in Study 1.

### Study 3

#### Procedure

Couples were recruited through our social acquaintances and in public places of Paris, Hyères, Grenoble and Montpellier in France. They are all Caucasian and have at least one child (a factor that we used to ensure that the couples were in long-term relationships at the time of the study). For each couple, the man and the woman were classified by three independent evaluators (AA, JB and MR). The five characteristics studied were the same as in Study 2: hair and eye color, chin dimple, and thickness of the lips and eyebrows.

#### Statistical analysis

For each trait, the association of the phenotypes of mates was tested using a one-sided Fisher’s exact test on a 2×2 contingency table, with homogamy as the alternative hypothesis. To generate a global test for the five traits, a joint distribution of the five tables was generated by 1000 resampling with the same marginal counts for each table. The product of the odd-ratio of each of the five-sample tables was used as a statistic. The proportion of these statistics that was higher than or equal to the observed value was used as an estimation of the *P*-value.

## Results

### Study 1

We quantified the attractiveness of young women using their facial pictures. We investigated whether men are more likely to prefer women with more recessive traits or with traits similar to their own.

#### Final sample

A total of 59 young women were sampled (age range: 18–25, mean = 22.4). Three hundred and sixty-one male raters assessed the relative attractiveness of these women. Assessments from unreliable raters (i.e., with more than one incorrect answer during the test of judgment reliability) were removed. A total of 345 raters were retained in the final sample, all Caucasian and heterosexual, with a mean age of 27.1 (age range: 18–61).

#### Importance of recessivity and homogamy

The relative importance (*I*) of each variable demonstrated with high probability that the recessivity index is in the best approximating model, with a negative effect (averaged parameter estimate β = −0.10; *I* >0.99) on the probability for a woman to be chosen. A majority of men preferred women with more dominant features. There was a positive effect (β = 0.04; *I* = 0.80) of the homogamy variable. To more precisely understand male preferences, the same indices were tested trait-by-trait in a secondary model. Although the dominant state of lips, eyes and earlobes were significantly preferred (*I* >0.99), there was a significant preference (*I* >0.99) for the recessive state of the eyebrows and chin dimple, and the thickness of lips was significantly preferred when homogamous (*I* = 0.65).

#### Importance of other women’s characteristics

Several variables demonstrated a significant probability to be in the best approximating model: Young (β = −0.28; *I* = 0.91) and more frequently smiling (*I* >0.99) women were more likely to be chosen by raters. The “asymmetry” variable was adjusted for using the function: × *+ exp(x).* More symmetric women were more often chosen (β = −0.04; *I* >0.99). The factor *exp(x)* indicates that attractiveness decreases faster than linearly with asymmetry. To determine the effects of femininity, the association between the degree of smiling and squared femininity was analyzed. Attractiveness increased with femininity, especially if the woman was smiling. Lip contrast did not exhibit a significant probability to be in the best approximating model (*I* = 0.49).

#### Effects of raters’ characteristics

The raters’ ages (*I* = 0.85) and socioeconomic levels (*I* = 0.61) exhibited a significant probability to be in the best approximating model.

### Study 2

To differentiate the effects of recessivity and homogamy from other predictors of attractiveness, we conducted an experiment similar to that performed in Study 1 using artificial faces.

#### Final Sample

One hundred and sixty-nine male raters expressed their preference. Assessments from unreliable raters (i.e., with more than one incorrect answer during the test of judgment reliability) were removed. Individuals with all the 5 features studied in the recessive (or dominant) state were also removed. A total of 99 raters were retained in the final sample, all Caucasian and heterosexual, with a mean age of 37.3 (age range: 19–84). A multinomial test was used on a sample of 1 response from each rater (randomly sampled for each rater among the fifteen women tested). This process was repeated 1,000 times, generating a distribution of *P*-values.

#### Homogamy and recessivity: general indices

**Figure 3:**
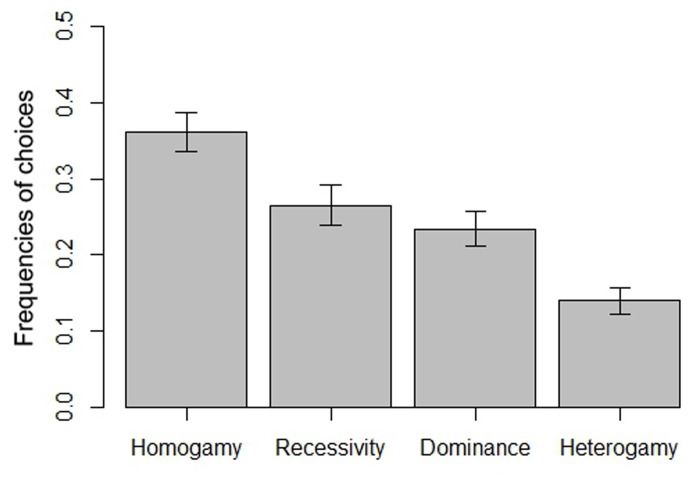
The raters' choices according to the artificial faces hypotheses in Study 2 (See figure 2). The raters predominantly preferred faces similar to their own.

The most frequent choice (37%, *P* = 0.036, see figure 3) tended to be the women presenting similar facial features as the rater. Thus, women presenting similar phenotypes as the raters themselves were predominantly preferred (homogamy). The multimodel inference on the binomial generalized linear mixed model showed that the homogamy index exhibited a significant probability to be in the best approximating model (*β* = 0.40; *I* >0.99) but not the recessivity index (*β* = 0.00; *I* = 0.27). The raters’ age exhibited a significant probability to be in the best approximating model (*I* >0.59). The raters’ choices were not influenced by SES (*I* = 0.29).

#### Homogamy and recessivity: analysis by facial trait

The same tendency toward homogamy was significant for all of the facial features analyzed individually: eye color (*β* = 1.12; *I* >0.99), hair color (*β* = 0.73; *I* >0.99), chin dimple (*β* = 0.98; *I* >0.99), and lip (*β* = 0.55; *I* >0.99) and eyebrow thickness (*β* = 1.29; *I* >0.99). We also found a considerable effect of the traits' absolute state. Raters exhibited a preference for the dominant state of two features: brown eyes (*β* = −0.21; *I* = 0.69) and dark hair (*β* = −0.47; *I* = 0.99). The recessive state was preferred for the three other features: chin dimple absence (*β* = 0.30; *I* = 0.82), thin lips (*β* = 0.26; *I* = 0.66) and eyebrows (*β* = 1.29; *I* >0.99). However, these effects might be the consequence of homogamy that is linked to unbalanced traits within the raters’ sample. For example, more than 80% of raters had brown hair, and they mainly preferred women with brown hair. This hypothesis was confirmed by separating raters according to their own traits (two groups for each characteristic). Preference for homogamy according to specific facial features appeared to best explain these results. Global preference toward homogamy remain unchanged even when quartet where some faces differed by only one trait were removed (corresponding to raters with almost all features in dominant or recessive state).

#### Predictions of paternity uncertainty hypothesis and the homogamy hypothesis

Consistent with the homomagmy hypothesis, phenotypically dominant men prefer phenotypically dominant women, and phenotypically recessive men prefer phenotypically recessive women (see table 2). Contrarily to the paternity uncertainty hypothesis, there is no support for a lower assortation for dominant traits compared to recessive traits. The only exception is for the eyebrows (see table 2), probably due to the fact that thin eyebrows are considered as more feminine, and so more attractive [1]. Thus phenotypically dominant men could be attracted by women with thin eyebrows too, limiting the homogamy effect for this specific feature.

**Table 2:** Table. 2. Testing the homogamy and the paternity uncertainty hypotheses, according to raters and women’s phenotypes.

	Homogamy			Paternity uncertainty
Traits	DD>RD	and	RR>DR	RR>DD
Eyes	**<10**−**5**		**<10**−**5**	0.73
Hair	**<10**−**5**		0.14	0.992
Chin	**0.00255**		**<10**−**5**	0.17
Lips	0.07		**0.00003**	0.28
Eyebrows	0.43		**<10**−**5**	**0.00015**
All	**<10**−**5**		**<10**−**5**	0.39

Predictions are coded as follows: the first letter indicates the phenotype of women preferred, and the second the rater’s phenotype. Thus “DD>RD” corresponds to the hypothesis “the preference of phenotypically dominant men towards phenotypically dominant women is stronger than the preference of phenotypically recessive men towards phenotypically dominant women”. *P*-value for each trait and overall are presented. Bold *P*-values highlight significant (*P*<0.05) results.

### Study 3

Here, we investigated whether the preferences observed during experiments are actually applied in real mate choice. We therefore determined whether the trends of facial homogamy identified in Studies 1 and 2 were significantly conserved in a sample of real couples. The analysis was based on 155 Caucasian couples. First, the partners were analyzed trait-by-trait using Fisher's exact tests. There were positive but non-significant associations (when corrected for multiple testing: confidence threshold = 0.05/5 tests = 0.01) for lip thickness (*OR* = 2.44, *P* = 0.023), eye color (*OR* = 1.38, *P* = 0.22), hair color (*OR* = 1.41, *P* = 0.35), and chin dimple (*OR* = 3.61, *P* = 0.45). There was also a negative and a non-significant association for eyebrow thickness (*OR* = 0.69, *P* = 0.89). Next, the combined 5 traits were analyzed together. A significant trend toward overall homogamy was revealed (*P* = 0.018). These results suggested that couple formation is driven by a global similarity rather than a homogamy for particular traits. It is also possible that men prefer women with all traits corresponding to their own but that the realization of these preferences during mate choice is incomplete and cannot be achieved for all facial features.

## Discussion

Although there is abundant evidence showing that cues indicating reproductive potential are considered attractive in women, few empirical studies have investigated the role of other selective pressures on men’s mate choice, particularly considering that choice is not absolute but is also relative to the men’s phenotype. This study investigates whether facial traits are more attractive if they are (i) recessive phenotypes, increasing the probability that subsequent offspring will exhibit a phenotype that facilitates paternal recognition (Salter's paternity uncertainty hypothesis [3]), and (ii) phenotypically similar to the facial traits of the rater (e.g., homogamy), which might indicate optimum genetic compatibility between mates. These hypotheses were tested on seven facial traits using both actual and virtual facial stimuli. The predicted preferences were then compared to realized mate choices using the facial pictures from couples with at least 1 child. Our data suggest that men prefer facial traits similar to their own, in both preference experiments and actual mate choice. Our results, however, do not support the hypothesis that men prefer women with traits that facilitate the detection of paternity/non-paternity in their offspring.

The raters of our study did not exhibit particular preferences for women’s recessive facial traits, contrary to the prediction of the paternity uncertainty hypothesis. Thus, women’s facial attractiveness does not appear to comprise a signature of paternity confidence, at least not as proposed by Salter [3]. An empirical knowledge of inheritance rules is required to discern whether mate preference can evolve according to the Salter hypothesis. Despite evidence for the common knowledge of inheritance [40], [41], several uncertainties prevent the application of this general knowledge to specific features. First, the low population frequency of some heritable features prevents the development of an intuitive comprehension of their mode of inheritance (e.g., freckles, red hair, white forelock). Second, non-heritable features, such as various types of spots, pimples, infantile haemangioma, angioma, result in a confounding background noise for any attempt to decipher how other specific traits are inherited. Third, the exact genetic determinism is often more complex than the simple Mendelian inheritance considered here, and a non-negligible environmental influence contributes to obscure the inheritance pattern [34]. Moreover, the instability of some facial traits in newborns compromises the reliability of those traits for the inference of paternity. For example, eye color, for which the recessive state (blue) is often first expressed in young babies (at least for Caucasian individuals), is replaced by a brown (or green or grey) color for those possessing the corresponding allele later in life [42]. Such is also the case for hair color, with a longer expression of the recessive state (blond). This could correspond to an anonymization of the baby, as the phenotypes of babies with those features tend to be similar –independently of their parents’ genotype. Indeed, children may benefit from anonymization in some circumstances (*i.e.*, in the case of non-paternity) [43]. It is known that such anonymization is parent-specific, as newborn babies tend to resemble their mothers [12], [44], [45], [46], but see [47], [48]. Why some features are partially anonymized whereas others are not remains an open question. There is the intriguing possibility that the process of anonymization could be restricted to traits that exhibit Mendelian inheritance because of the constraints associated with traits coded by many genes. This, in turn, decreases the extent of selection that assesses paternity confidence based on traits that exhibit Mendelian inheritance.

This study provides evidence for the homogamy for certain facial features. The male raters in this study preferred women’s faces that exhibited the same states as themselves for five out of the seven traits investigated (eye color, hair color, chin dimple, thickness of lips and eyebrows). This finding was revealed when artificial faces were used (Study 2) and only partially revealed (thickness of lips) when faces from real women were used (Study 1). Although many dimensions comprise facial attractiveness, only two have been controlled for in experiment 1 (overall femininity and symmetry). Men have been previously demonstrated to prefer the most attractive female faces rather than self-resembling ones, although when attractiveness was controlled for, the self-resembling faces were preferred [49]. This probably explains the discrepancy between the results of Study 1 (where two attractiveness components were statistically controlled for) and Study 2 (where all attractiveness components were experimentally controlled for). To avoid false statistical associations among traits that might result from the numerous dimensions involved in facial attractiveness, a very large sample size is required. In a limited sample, an apparent preference for a trait can arise indirectly: it could reflect a preference for another trait associated by chance within the sample. Conversely, Study 2 used a protocol that adjusts artificial faces of the experiment to each rater's face, while controlling for non-relevant variables. A limitation of Study 1 is that the question asked to the raters (“which of the 2 women is more attractive?”), was not explicitly proposed in a context of long or short-term relationship, which can influence preferences [50].

Why would self-resembling mates be preferred? When resemblance is based on a large number of genetically heritable traits, it becomes a measure of genetic proximity. Due to the known inbreeding depression in most human societies [51], [52], [53], [54], mate preference based on high resemblance (i.e., toward close kin) should be avoided. Indeed, self-resemblance decreases attractiveness in a short-term sexual context [55]. This avoidance is in fact enforced by a biological mechanism (Westermark effect) that suppresses sexual attraction toward co-residents during childhood–generally siblings or close members of kin [56], [57], [58], [59]. This biological mechanism is further reinforced by social rules forbidding incestuous marriages, where the definition of incest is based on family linkage (with a large cultural variation) [60]. Considering these mechanisms enforcing avoidance of inbreeding depression, some degree of homogamous mate preference should develop only if there is also an extent outbreeding depression. Outbreeding can induce a loss of fitness in several species [61], [62]. Outbreeding depression can be attributed to the disruption of local adaptation (cultural or genetic), underdominance, or epistatic interactions. Ideally, there should be an optimal balance between inbreeding and outbreeding [63]. Interestingly, in Iceland, the greatest reproductive success is for couples related at the level of third and fourth cousins, with a decrease in fertility for closer or more distantly related couples [23]. It is thus possible that homogamy results from a selection for avoiding outbreeding depression, given that inbreeding is already prevented by other mechanisms (biological and social).

The homogamy hypothesis is possibly confounded by the familial imprinting effect e.g. preference for the face of the opposite-sex parent [24], [64], [65]. However, because children resemble their parents, it’s difficult to distinguish between effects of familial imprinting and homogamy (self-referential phenotype matching). Clearly, there is also the possibility that preference for the face of the opposite-sex parent correspond to a preference for homogamous mating. Nevertheless, it seems that these two mechanisms exist, and have context-specific effects [66].

Mate preference does not readily translate into actual mate choice, due to mate competition and social subdivisions. It is even possible that traits considered to significantly influence attractiveness do not exert effective influence on actual pairing [29], suggesting that preferences might not always be readily deduced from observed pairing. The observed homogamy in real couples for the five facial features examined in our study suggests that preference for homogamous mating has operated. There are, however, several potentially confounding phenomena that might account for apparently homogamous mating. For instance, if there is competition in both sexes for long-term mates, a similarity between romantic partners would result as a side effect [67]. Attractive individuals might pick their own preferred (and attractive) mate from the pool of those available, leaving less attractive individuals to pair with those that remain unpaired. Thus, homogamy might result from such a process, despite the fact that all individuals prefer the same category (attractiveness) for mates. However, as raters in this study did not prefer the same features (their preferences depended on their own traits), this process is unlikely to apply here. Similarly, resemblance could result from long-term environment sharing, rather than from preferences at the time of couple formation [5], [6], [25]. However, the traits considered here mainly have a genetic basis and are thus not particularly prone to such effects. Homogamy might also result from social proximity. Indeed, people are more likely to meet their mate within their own social class; thus, there is a high probability that they will share traits that are specific to this class. A prerequisite for this hypothesis is that the features involved are differently distributed across social classes. To our knowledge, the distribution of facial features across social classes has not been studied.

Homogamy in the context of the facial features examined in our study has already been reported for hair color (0.28) and eye color (0.21) [20], [68]. Concerning preferences, Laeng et al. [20] found that, consistent with our study, blue-eyed men tended to rate blue-eyed women as more attractive than brown-eyed ones. However, they also found that brown-eyed men exhibited no preference for blue- or brown-eyed women. In contrast, we observed that brown-eyed men found brown eyes women more attractive. The reasons for this discrepancy are unclear and might originate from differences in experimental design (e.g., manipulated photographs of real faces versus manipulated artificial faces; attractiveness scale versus choice among alternatives), true cultural differences (e.g., Norway versus southern France) or random effects (e.g., the number of raters was about 4-fold larger in the present study). Future studies are warranted to clarify this point.

Women’s facial attractiveness is complex and includes many components. Paternity signaling is not a significant component of attractiveness, at least within the experimental context of this study. Mate similarity, although certainly not a predominant component compared to fertility cues, is likely a component that should be thoroughly controlled for in future studies of homogamous preference. Further studies are necessary to verify whether the preference for homogamous traits is favored as a way to avoid outbreeding and its associated fitness costs. Although these costs have been delineated in many other species, they remain to be investigated in humans. Finally, the underlying mechanisms of outbreeding depression need to be better understood.

## Supporting Information

Figure S1 **Points for measurement of asymmetry and femininity.** For asymmetry, eight distances on each side of the face were considered: (2-3) vs (4-5), (15-17) vs (18-20), (16-8) vs (19-9) and (3-21) vs (4-21). For femininity/masculinity, nine distances were computed: (2-5), (3-4), (6-7), (8-9), (11-12), (10-13), (2-14), (1-14) and (11-14).
(TIF)

Figure S2 **Linear discriminant analysis (LDA) according to sex.** It provides a synthetic variable corresponding to a masculinity/femininity axis. The coordinate of each woman along this axis represents her individual femininity value.
(TIF)

## References

[1] Rhodes G (2006) The evolutionary psychology of facial beauty. Annu Rev Psychol 57: 199–226. 16318594 10.1146/annurev.psych.57.102904.190208

[2] Law-Smith MJ, Perrett DI, Jones BC, Cornwell RE, Moore FR, et al (2006) Facial appearance is a cue to oestrogen levels in women. Proc R Soc Lond B Biol Sci 273: 135–140. 10.1098/rspb.2005.3296 PMC1560017 16555779

[3] Salter F (1996) Carrier females and sender males: An evolutionary hypothesis linking female attractiveness, family resemblance, and paternity confidence. Ethol Sociobiol 17: 211–220.

[4] Penton-Voak IS, Perrett DI, Peirce JW (1999) Computer graphic studies of the role of facial similarity in judgements of attractiveness. Curr Psychol 18: 104–117.

[5] Hinsz VB (1989) Facial resemblance in engaged and married couples. J Soc Pers Relat 6: 223–229.

[6] Keller MC, Thiessen D, Young RK (1996) Mate assortment in dating and married couples. Pers Individ Dif 21: 217–221.

[7] Grafen A (1990) Do animals really recognize kin? Anim Behav 39: 42–54.

[8] Platek SM, Burch RL, Panyavin IS, Wasserman BH, Gallup GG Jr (2002) Reactions to children's faces: Resemblance affects males more than females. Evol Hum Behav 23: 159–166.

[9] DeBruine LM (2004) Resemblance to self increases the appeal of child faces to both men and women. Evol Hum Behav 25: 142–154.

[10] Apicella CL, Marlowe FW (2004) Perceived mate fidelity and paternal resemblance predict men's investment in children. Evol Hum Behav 25: 371–378.

[11] Alvergne A, Faurie C, Raymond M (2009) Father-offspring resemblance predicts paternal investment in humans. Anim Behav 78: 61–69.

[12] Alvergne A, Faurie C, Raymond M (2010) Are parents' perceptions of offspring facial resemblance consistent with actual resemblance? Effects on parental investment. Evol Hum Behav 31: 7–15.

[13] Burch RL, Gallup GG Jr (2000) Perceptions of paternal resemblance predict family violence. Evol Hum Behav 21: 429–435. 11146307 10.1016/s1090-5138(00)00056-8

[14] Welling LLM, Burriss RP, Puts DA (2010) Mate retention behavior modulates men's preferences for self-resemblance in infant faces. Evol Hum Behav 32: 118–126.

[15] Lacy RC, Sherman PW (1983) Kin recognition by phenotype matching. Am Nat 121: 489–512.

[16] Davenport GC, Davenport CB (1907) Heredity of eye color in man. Science 26: 589–592. 17754423 10.1126/science.26.670.589-b

[17] Eiberg H, Troelsen J, Nielsen M, Mikkelsen A, Mengel-From J, et al (2008) Blue eye color in humans may be caused by a perfectly associated founder mutation in a regulatory element located within the HERC2 gene inhibiting OCA2 expression. Hum Genet 123: 177–187. 18172690 10.1007/s00439-007-0460-x

[18] Sturm RA, Larsson M (2009) Genetics of human iris colour and patterns. Pigment Cell Melanoma Res 22: 544–562. 19619260 10.1111/j.1755-148X.2009.00606.x

[19] Duffy DL, Montgomery GW, Chen W, Zhao ZZ, Le L, et al (2007) A three-single-nucleotide polymorphism haplotype in intron 1 of OCA2 explains most human eye-color variation. Am J Hum Genet 80: 241–252. 17236130 10.1086/510885 PMC1785344

[20] Laeng B, Mathisen R, Johnsen JA (2007) Why do blue-eyed men prefer women with the same eye color? Behav Ecol Sociobiol 61: 371–384.

[21] Prokop P, Obertova Z, Fedor P (2010) Paternity cues and mating opportunities: what makes fathers good? Acta Ethol 13: 101–107.

[22] Rushton JP (1989) Genetic similarity, human altruism, and group selection. Behav Brain Sci 12: 503–559.

[23] Helgason A, Snaebjörn P, Guobjartsson DF, Kristjansson P, Stefansson K (2008) An association between the kinship and fertility of human couples. Science 319: 813–816. 18258915 10.1126/science.1150232

[24] Bereczkei T, Gyuris P, Koves P, Bernath L (2002) Homogamy, genetic similarity, and imprinting; parental influence on mate choice preferences. Pers Individ Dif 33: 677–690.

[25] Little AC, Burt DM, Perrett DI (2006) Assortative mating for perceived facial personality traits. Pers Individ Dif 40: 973–984.

[26] Dribe M, Lundh C (2009) Status homogamy in the preindustrial marriage market: Partner selection according to age, aocial origin, and place of birth in nineteenth-century rural Sweden. J Fam Hist 34: 387–406. 19999643 10.1177/0363199009344708

[27] Hartung A, Vandezande V, Phalet K, Swyngedouw M (2011) Partnership preferences of the Belgian second generation: Who lives with whom? Adv Life Course Res 16: 152–163.

[28] Solis P, Pullum TW, Bratter J (2007) Homogamy by education and migration status in Monterrey, Mexico: changes and continuities over time. Popul Res Policy Rev 26: 279–298.

[29] Courtiol A, Picq S, Godelle B, Raymond M, Ferdy JB (2010) From Preferred to Actual Mate Characteristics: The Case of Human Body Shape. PLoS One 5. 10.1371/journal.pone.0013010 PMC2946385 20885953

[30] Lebow MR, Savwin PB (1941) Inheritance of facial features: a pedigree study involving length of face, prominent ears and chin cleft. J Hered 32: 127–132.

[31] Bhanu V, Malhotra KC (1972) Population genetic study of cleft chin in India. Am J Phys Anthropol 37: 367–372. 5082930 10.1002/ajpa.1330370306

[32] Dutta P, Ganguly P (1965) Further observations on ear lobe attachment. Acta Genet Stat Med 15: 77–86. 14277139 10.1159/000151894

[33] Bastiaens M, ter Huurne J, Gruis N, Bergman W, Westendorp R, et al (2001) The melanocortin-1-receptor gene is the major freckle gene. Hum Mol Genet 10: 1701–1708. 11487574 10.1093/hmg/10.16.1701

[34] Susanne C (1975) Genetic and Environmental Influences on Morphological Characteristics. Ann Hum Biol 2: 279–287. 16431681 10.1080/03014467500000851

[35] Little AC, Jones BC, Waitt C, Tiddeman BP, Feinberg DR, et al (2008) Symmetry is related to sexual dimorphism in faces: Data across culture and species. PLoS One 3: e2106. 18461131 10.1371/journal.pone.0002106 PMC2329856

[36] Penton-Voak IS, Jones BC, Little AC, Baker S, Tiddeman B, et al (2001) Symmetry, sexual dimorphism in facial proportions and male facial attractiveness. Proc R Soc Lond B Biol Sci 268: 1617–1623. 10.1098/rspb.2001.1703 PMC1088785 11487409

[37] Burnham KP, White GC (2002) Evaluation of some random effects methodology applicable to bird ringing data. J Appl Stat 29: 245–264.

[38] Symonds MRE, Moussalli A (2011) A brief guide to model selection, multimodel inference and model averaging in behavioural ecology using Akaike's information criterion. Behav Ecol Sociobiol 65: 13–21.

[39] Hegyi G, Garamszegi LZ (2011) Using information theory as a substitute for stepwise regression in ecology and behavior. Behav Ecol Sociobiol 65: 69–76.

[40] Springer K, Keil FC (1989) On the development of biologically specific beliefs - the case of inheritance. Child Dev 60: 637–648. 2737013

[41] Richards M, Ponder M (1996) Lay understanding of genetics: A test of a hypothesis. J Med Genet 33: 1032–1036. 9004138 10.1136/jmg.33.12.1032 PMC1050817

[42] Matheny AP, Dolan AB (1975) Changes in eye color during early-childhood - Sex and genetic differences. Ann Hum Biol 2: 191–196. 1053245 10.1080/03014467500000731

[43] Pagel M (1997) Desperately concealing father: A theory of parent-infant resemblance. Anim Behav 53: 973–981.

[44] Nesse RM, Silverman A, Bortz A (1990) Sex-Differences in Ability to Recognize Family Resemblance. Ethol Sociobiol 11: 11–21.

[45] McLain DK, Setters D, Moulton MP, Pratt AE (2000) Ascription of resemblance of newborns by parents and nonrelatives. Evol Hum Behav 21: 11–23.

[46] Alvergne A, Faurie C, Raymond M (2007) Differential resemblance of young children to their parents: Who do children look like more. Evol Hum Behav 28: 135–144.

[47] Bredart S, French RM (1999) Do babies resemble their fathers more than their mothers? A failure to replicate Christenfeld and Hill (1995). Evol Hum Behav 20: 129–135.

[48] Bressan P, Grassi M (2004) Parental resemblance in 1-year-olds and the Gaussian curve. Evol Hum Behav 25: 133–141.

[49] Kocsor F, Rezneki R, Juhasz S, Bereczkei T (2011) Preference for facial self-resemblance and attractiveness in human mate choice. Arch Sex Behav 40: 1263–1270. 21267643 10.1007/s10508-010-9723-z

[50] Geary D, Vigil J, Byrd-Craven J (2004) Evolution of Human Mate Choice. J Sex Res 41: 27–42. 15216422 10.1080/00224490409552211

[51] Postma E, Martini L, Martini P (2010) Inbred women in a small and isolated Swiss village have fewer children. J Evol Biol 23: 1468–1474. 20492085 10.1111/j.1420-9101.2010.02013.x

[52] Das BK (2005) Inbreeding depression in anthropometric traits among Telaga boys of Kharagpur, West Bengal, India. Coll Antropol 29: 459–464. 16417144

[53] Jorde LB (2001) Consanguinity and prereproductive mortality in the Utah Mormon population. Hum Hered 52: 61–65. 11474206 10.1159/000053356

[54] Bittles AH (2001) Consanguinity and its relevance to clinical genetics. Clin Genet 60: 89–98. 11553039 10.1034/j.1399-0004.2001.600201.x

[55] DeBruine LM (2005) Trustworthy but not lust-worthy: context-specific effects of facial resemblance. Proc R Soc Lond B Biol Sci 272: 919–922. 10.1098/rspb.2004.3003 PMC1564091 16024346

[56] Wolf AP (1970) Childhood association and sexual attraction - a further test of Westermarck hypothesis. Am Anthropol 72: 503–515.

[57] Vandenberghe PL, Mesher GM (1980) Royal incest and inclusive fitness. Am Ethnol 7: 300–317.

[58] Walter A, Buyske S (2003) The Westermarck effect and early childhood co-socialization: Sex differences in inbreeding-avoidance. Br J Dev Psychol 21: 353–365.

[59] Lieberman D, Tooby J, Cosmides L (2007) The architecture of human kin detection. Nature 445: 727–731. 17301784 10.1038/nature05510 PMC3581061

[60] Bittles AH, Mason WM, Greene J, Rao NA (1991) Reproductive-behavior and health in consanguineous marriages. Science 252: 789–794. 2028254 10.1126/science.2028254

[61] Keller LF, Waller DM (2002) Inbreeding effects in wild populations. Trends Ecol Evol 17: 230–241.

[62] Edmands S (2007) Between a rock and a hard place: evaluating the relative risks of inbreeding and outbreeding for conservation and management. Mol Ecol 16: 463–475. 17257106 10.1111/j.1365-294X.2006.03148.x

[63] Bateson P (1978) Sexual imprinting and optimal outbreeding. Nature 273: 659–660. 661972 10.1038/273659a0

[64] Little AC, Penton-Voak IS, Burt DM, Perrett DI (2003) Investigating an imprinting-like phenomenon in humans Partners and opposite-sex parents have similar hair and eye colour. Evol Hum Behav 24: 43–51.

[65] Bereczkei T, Gyuris P, Weisfeld GE (2004) Sexual imprinting in human mate choice. Proc R Soc Lond B Biol Sci 271: 1129–1134. 10.1098/rspb.2003.2672 PMC1691703 15306362

[66] Watkins CD, DeBruine LM, Smith FG, Jones BC, Vukovic J, et al (2011) Like father, like self: emotional closeness to father predicts women's preferences for self-resemblance in opposite-sex faces. Evol Hum Behav 32: 70–75.

[67] Miller GF, Todd PM (1998) Mate choice turns cognitive. Trends Cogn Sci 2: 190–198. 21227154 10.1016/s1364-6613(98)01169-3

[68] Russell RJH, Wells PA, Rushton JP (1985) Evidence for genetic similarity detection in human marriage. Ethol Sociobiol 6: 183–187.

