# Supplementary figures and images for: Men’s Preference for Women’s Facial Features: Testing Homogamy and the Paternity Uncertainty Hypothesis
Source: PLoS One. 2012 Nov 21;7(11):e49791. doi: 10.1371/journal.pone.0049791 (PMC3504097; doi:10.1371/journal.pone.0049791)

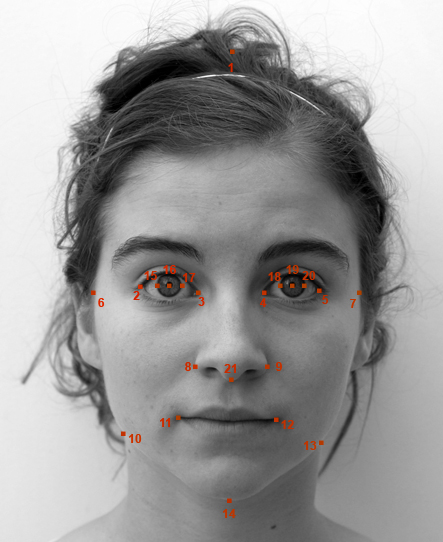

Supplement: Figure S1 — Points for measurement of asymmetry and femininity. For asymmetry, eight distances on each side of the face were considered: (2-3) vs (4-5), (15-17) vs (18-20), (16-8) vs (19-9) and (3-21) vs (4-21). For femininity/masculinity, nine distances were computed: (2-5), (3-4), (6-7), (8-9), (11-12), (10-13), (2-14), (1-14) and (11-14). (TIF) [file pone.0049791.s001.tif]

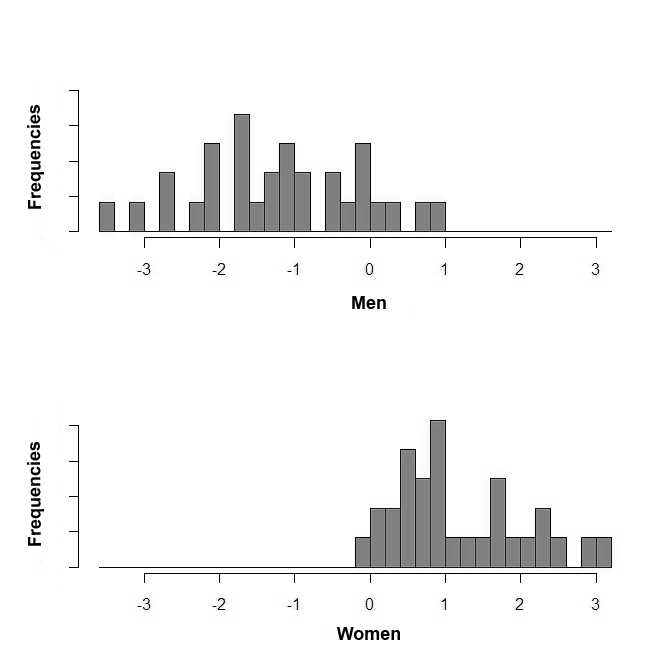

Supplement: Figure S2 — Linear discriminant analysis (LDA) according to sex. It provides a synthetic variable corresponding to a masculinity/femininity axis. The coordinate of each woman along this axis represents her individual femininity value. (TIF) [file pone.0049791.s002.tif]
